# The progress and prospects of targeting the adenosine pathway in cancer immunotherapy

**DOI:** 10.1186/s40364-025-00784-0

**Published:** 2025-05-19

**Authors:** Yuying Yang, Lin Zhu, Yantao Xu, Long Liang, Li Liu, Xiang Chen, Hui Li, Hong Liu

**Affiliations:** 1 Department of Dermatology, Hunan Engineering Research Center of Skin Health and Disease, Hunan Key Laboratory of Skin Cancer and Psoriasis, Xiangya Hospital, Central South University, Changsha, Hunan 410008 China; 2 National Engineering Research Center of Personalized Diagnostic and Therapeutic Technology, Central South University, Changsha, Hunan 410008 China; 3 National Clinical Research Center for Geriatric Disorders, Xiangya Hospital, Central South University, Changsha, Hunan 410008 China; 4 Molecular Biology Research Center and Center for Medical Genetics, School of Life Sciences, Central South University, Changsha, Hunan 410078 China

**Keywords:** Adenosine, Cancer immunotherapy, CD73, CD39, Adenosine receptors, Tumor microenvironment

## Abstract

Despite the notable success of cancer immunotherapy, its effectiveness is often limited in a significant proportion of patients, highlighting the need to explore alternative tumor immune evasion mechanisms. Adenosine, a key metabolite accumulating in hypoxic tumor regions, has emerged as a promising target in oncology. Inhibiting the adenosinergic pathway not only inhibits tumor progression but also holds potential to enhance immunotherapy outcomes. Multiple therapeutic strategies targeting this pathway are being explored, ranging from preclinical studies to clinical trials. This review examines the complex interactions between adenosine, its receptors, and the tumor microenvironment, proposing strategies to target the adenosinergic axis to boost anti-tumor immunity. It also evaluates early clinical data on pharmacological inhibitors of the adenosinergic pathway and discusses future directions for improving clinical responses.

## Introduction

The idea of utilizing the immune system to combat cancer dates back to the early nineteenth century. However, the field of cancer immunotherapy experienced a renaissance in the past decade, particularly with the advent of checkpoint blockade therapy [1–7]. Immune checkpoints are cell-surface proteins that regulate the initiation, duration, and intensity of immune responses [8]. Notable examples of T-cell immune checkpoint molecules include cytotoxic T-lymphocyte antigen 4 (CTLA-4) and programmed cell death protein-1 (PD-1). The US FDA has approved single-agent checkpoint blockade or combination therapies targeting these molecules for an expanding array of malignancies [8–12]. Despite the effectiveness of immune checkpoint therapies in treating advanced cancer, a considerable number of patients remain unresponsive, suggesting the existence of additional immunosuppressive mechanisms within the tumor microenvironment (TME) [13–20]. One such mechanism is the adenosinergic pathway, which has emerged as a promising therapeutic target.

The immunosuppressive properties of extracellular adenosine are well-established, supporting the rationale for targeting this pathway in cancer immunotherapy. Nevertheless, adenosine's biological functions extend beyond immunomodulation, including neurodegeneration, nociception, vasodilation, and angiogenesis. These diverse roles emphasize the complexity of its signaling in both physiological and pathological contexts [21, 22]. A comprehensive understanding of adenosine's multifaceted role is essential for fully assessing the potential benefits and limitations of targeting adenosinergic pathways in oncology.

Under hypoxic conditions in tumors, oxygen deprivation triggers the accumulation of extracellular ATP (eATP), which primarily activates the immune response via P2 purinergic receptors [23–25]. eATP is then gradually degraded to adenosine, which modulates immune cell infiltration and activation by binding to P1 purinergic receptors, such as A1R, A2AR, A2BR, and A3R. Among these, adenosine-induced immunosuppression is primarily mediated by A2AR and A2BR receptors, leading to increased intracellular cAMP levels [26, 27]. Inhibition of adenosine-generating enzymes or receptors has shown promise in enhancing antitumor immune responses through various mechanisms. Clinical trials targeting the adenosinergic pathway in cancer patients are progressing rapidly.

This review examines adenosine metabolism and explores its potential as a target for cancer immunotherapy. An original pan-cancer analysis of genetic and epigenetic alterations in the adenosine pathway reveals variability in the dysregulation of CD39, CD73, A2AR, and A2BR across different cancers. Single-cell RNA-seq data from diverse tissues are analyzed to identify cell-type-specific expression patterns of adenosine signaling molecules in vivo. These observations clarify adenosine-induced immunosuppressive mechanisms within the TME and provide new insights into how tumor-intrinsic alterations in the adenosine pathway contribute to immune evasion. Additionally, the review discusses the current landscape of clinical trials targeting the adenosinergic pathway and explores the potential of combining adenosine pathway inhibition with other immunotherapies. It addresses mechanisms of resistance to adenosine blockade and examines predictive biomarkers for adenosine-targeted treatments. Finally, novel strategies are proposed to enhance immune responses.

## An overview of adenosine metabolism

**Fig. 1:**
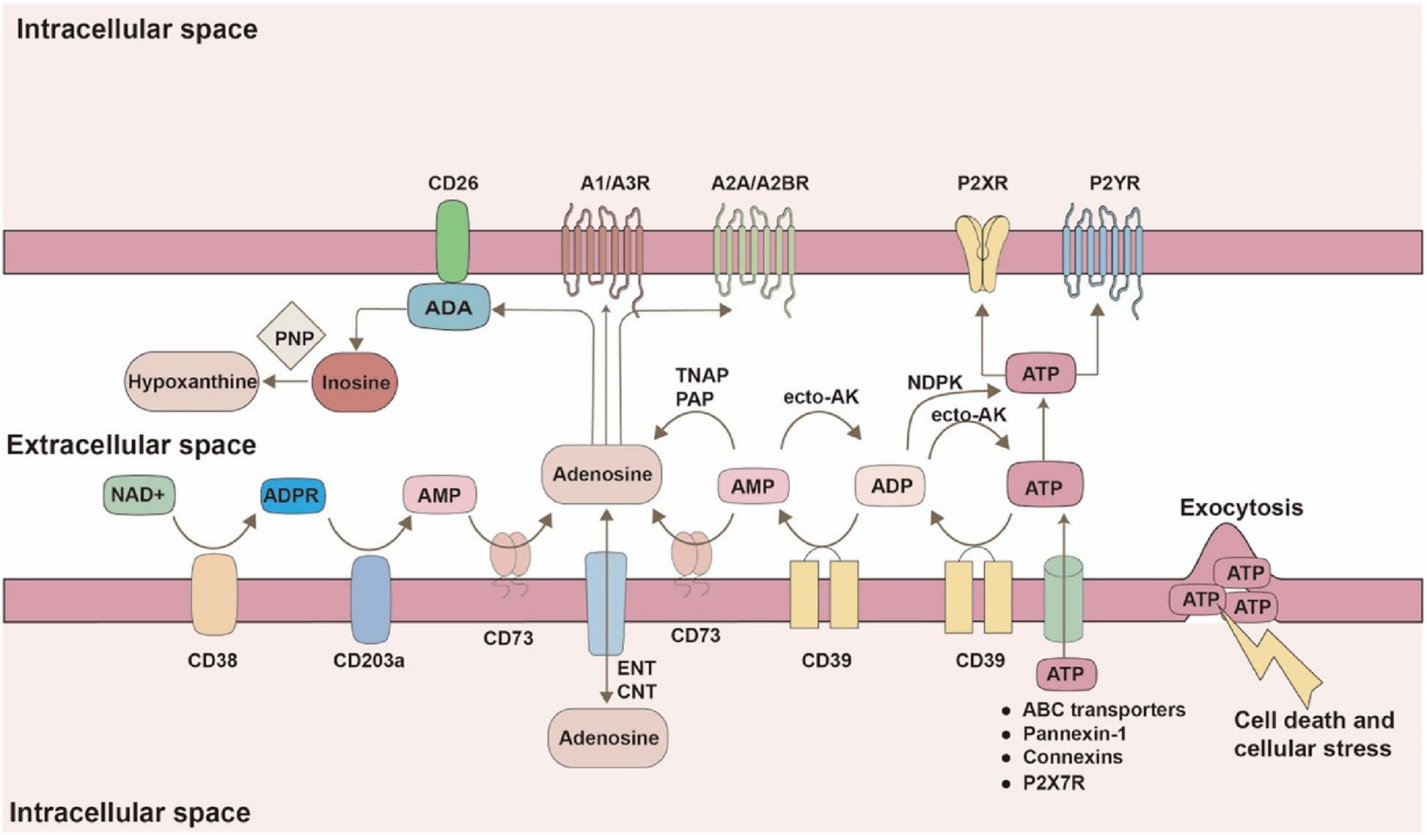
Adenosine production and signaling pathway. Following cell death or cellular stress, ATP is rapidly released into the extracellular space through mechanisms such as vesicle exocytosis, ABC transporters, pannexin-1, connexins, and P2X7R. Extracellular ATP can then activate P2X and P2Y receptors or be converted into adenosine via the ectonucleotidases CD39 and CD73. The enzymatic action of CD39 can be reversed by AK and NDPK. Adenosine can also be produced via the CD38-CD203a-CD73 pathway. In addition to ectonucleotidases, alternative membrane-bound phosphatases, including TNAP and PAP, can contribute to adenosine generation. Once generated, extracellular ADO can bind to P1 receptors (A1R, A2AR, A2BR, and A3R), be degraded to inosine by ADA, or be transported into the intracellular space through equilibrative or concentrative nucleoside transporters (ENTs and CNTs, respectively). ATP, adenosine triphosphate; ADP, adenosine diphosphate; AMP, adenosine monophosphate; ABC, ATP-binding cassette; AK, adenylate kinase; NDPK, nucleoside diphosphate kinase; TNAP, tissue-non-specific alkaline phosphatase; PAP, prostatic acid peptidase; ADO, adenosine; ADA, adenosine deaminase; PNP, purine nucleoside phosphorylase

Under homeostatic conditions, eATP levels are minimal. However, during cellular stress induced by hypoxia, ischemia, or inflammation, ATP is rapidly released into the extracellular space through mechanisms such as vesicle exocytosis, ATP-binding cassette (ABC) transporters, anion-selective channels, or non-selective pores formed by pannexin-1, connexins, and the ATP receptor P2X7R [23, 28]. eATP serves as a key "find me" signal, recruiting monocytes to the inflammation site [29]. Despite its potent immunostimulatory role in the extracellular environment, eATP is rapidly converted to adenosine through a stepwise hydrolysis process, facilitated by plasma membrane-expressed enzymes, including ecto-5'-nucleotidase (CD73), ectonucleoside triphosphate diphosphohydrolases (E-NTPDases), and ectonucleotide pyrophosphatase/phosphodiesterases (ENPPs) [30, 31] (Fig. 1).

The CD39-CD73 axis is the primary pathway for extracellular adenosine (eADO) production [32]. CD39 (known as ENTPD1), a member of the E-NTPDase family, catalyzes the conversion of extracellular ATP or ADP to AMP, a rate-limiting step, which is subsequently dephosphorylated to adenosine by CD73 [33]. While the CD39-CD73 pathway is the most well-characterized mechanism of adenosine generation, alternative, non-canonical pathways also contribute to extracellular adenosine production [34]. For instance, CD38 (an NAD^+^ nucleosidase) utilizes NAD^+^ as a substrate to generate adenosine diphosphate ribose (ADPR), which is subsequently converted into AMP by CD203a [35]. AMP is further hydrolyzed to adenosine by CD73 (Fig. 1). Interestingly, CD203a and CD203c (also known as ENPP3) can directly hydrolyze ATP to AMP, suggesting a potential compensatory role for CD39 [36, 37]. Under hypoxic conditions, the CD38-CD203a-CD73 pathway is believed to be more efficient, consuming high NAD^+^ levels in favor of the CD203a-CD73 axis. Additionally, adenosine can be generated through other membrane-bound AMP ectonucleotidases, such as tissue-non-specific alkaline phosphatases (TNAPs) and prostatic acid phosphatases (PAPs) [38].

Notably, ATP can be resynthesized in the extracellular space via adenylate kinase (AK) or nucleoside diphosphate kinase (NDPK) through phosphotransfer reactions [39, 40]. AK catalyzes the reversible phosphoryl transfer between ATP, ADP, and AMP, enabling the interconversion of these nucleotides (ATP + AMP ⇄ 2 ADP) [41]. This reaction not only regenerates ATP but also helps regulate the local purine pool. NDPK, responsible for exchanging phosphate groups between nucleoside diphosphates and triphosphates, maintains purine nucleotide homeostasis [42]. Both AK and NDPK play key roles in the local scavenging of extracellular nucleotides and may represent novel mechanisms for supplying substrates to ectoenzymes like CD39 and CD203a [43].

Two distinct classes of adenosine transporter proteins-equilibrative nucleoside transporters (ENTs) and concentrative nucleoside transporters (CNTs)-mediate extracellular adenosine uptake into the cytosol [44, 45]. ENTs enable bidirectional adenosine translocation, maintaining adenosine equilibrium across the cell membrane, while CNTs actively transport adenosine against its concentration gradient, ensuring higher intracellular adenosine levels [46]. Once internalized by cells via ENTs or CNTs, adenosine undergoes phosphorylation to ATP or deamination to inosine. This process reflects the dynamic interaction between extracellular and intracellular purine metabolism, precisely regulating cellular ATP and adenosine concentrations [41].

Excessive eADO can also be deaminated on the cell surface by ecto-adenosine deaminase (ADA), resulting in the production of inosine [47], which is subsequently converted to hypoxanthine by purine nucleoside phosphorylase (PNP) [48]. Thus, the concentration of eADO is tightly regulated not only by adenosine-producing enzymes but also by mechanisms involving ATP-regenerating pathways, nucleoside transporters, and adenosine-degrading enzymes, highlighting the redundancy and interdependence of ectoenzymatic and intracellular processes.

Extracellular adenosine exerts its regulatory effects by binding to the P1 receptors, a family of G-protein-coupled receptors (GPCRs) comprising A1R, A2AR, A2BR, and A3R [49, 50]. These receptors exhibit varying affinities for adenosine. A1R and A2AR, as high-affinity receptors, are sensitive to physiological adenosine levels and are highly expressed on cell surfaces (hA_1_*K*_i_ = 310 nM, hA_2 A_*K*_i_ = 700 nM) [51]. In contrast, A3R shows moderate to low affinity for adenosine [52], which is influenced by factors such as receptor expression levels, ligand concentration, and interactions with other receptor subtypes [53]. Additionally, A3R can form heterodimers with other adenosine receptors, especially A2A, potentially altering their pharmacological and signaling profiles [54]. Thus, the affinity of A3R for adenosine is context-dependent. A2BR, on the other hand, requires higher adenosine concentrations for activation (hA_2B_K_i_ ≥ 10 μM), typically seen under pathological conditions, such as within the TME [51, 55]. Adenosine receptor activation regulates adenylate cyclase (AC) activity, influencing the intracellular levels of cyclic AMP (cAMP) and downstream signaling pathways [56].

eATP exerts its effects by binding to P2 receptors expressed on both tumor and host cells, including the ionotropic P2X receptors (P2XR) and metabotropic P2Y receptors (P2YR) [57–59]. Activation of P2XRs, particularly P2X7 on immune cells, triggers the release of pro-inflammatory cytokines, such as IL-18 [58]. P2YRs, as classical GPCRs, initiate downstream signaling via specific receptor/Gα combinations.

## The expression patterns of adenosine signaling in cancer

The TME constitutes a complex ecosystem that fosters chronic inflammation, immunosuppression, and pro-angiogenesis, thereby promoting tumor growth and metastasis [60–63]. Under hypoxic conditions, ATP is rapidly released into the extracellular space, where it is converted to adenosine. Extracellular adenosine binds to P1 purinergic receptors, inhibiting immune responses. Within this environment, both immune and non-immune cells express functional adenosine-generating enzymes and adenosine receptors. Emerging evidence highlights adenosine as a pivotal mediator of tumor progression. In hepatocellular carcinoma, adenosine promotes tumor cell proliferation by regulating the cell cycle, driving aggressive growth [64]. Additionally, adenosine accumulation in melanoma enhances metastasis by promoting angiogenesis and immune evasion [65, 66]. In glioblastoma, adenosine further contributes to immune evasion by reprogramming macrophage polarization toward a pro-tumorigenic phenotype [67].

**Fig. 2:**
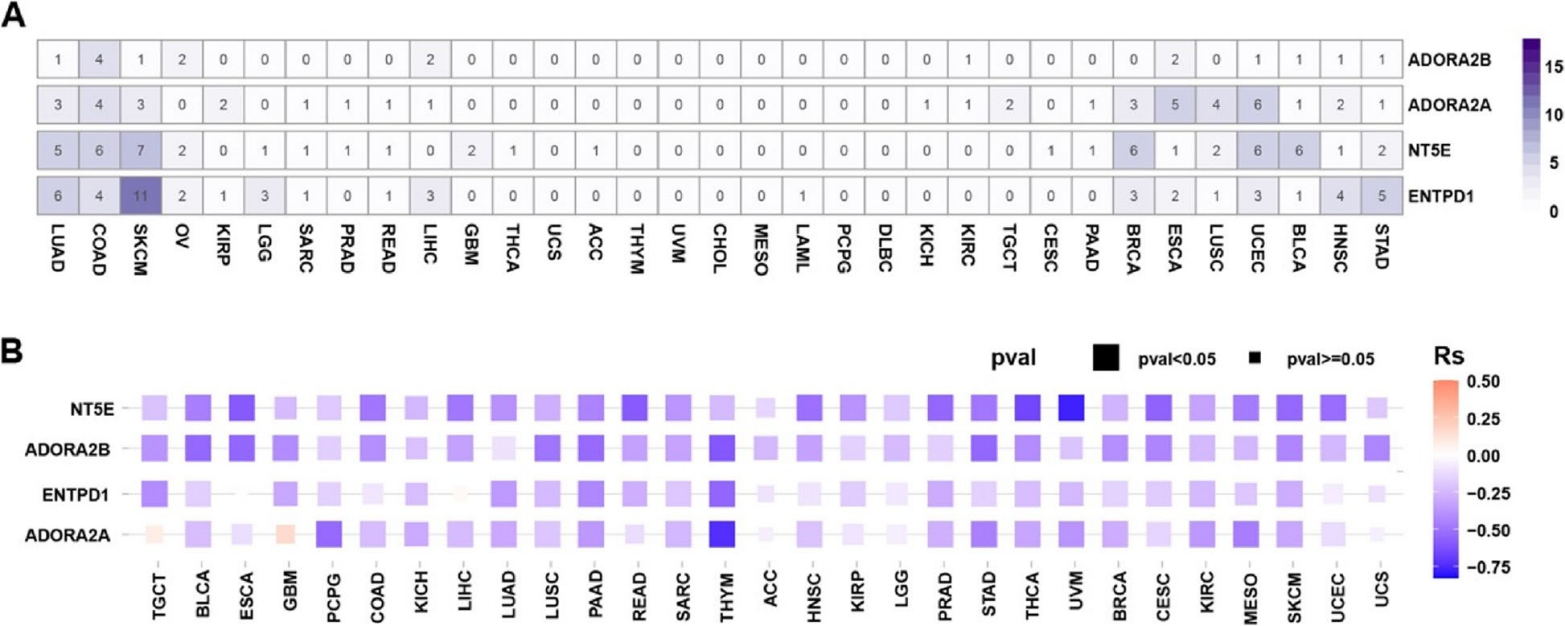
Mutation and methylation of CD73, CD39, A2AR, and A2BR in the adenosine signaling pathway. The Cancer Genome Atlas (TCGA) analysis of mutation (**A**) and methylation (**B**) data for NT5E, ENTPD1, ADORA2A and ADORA2B, which encode the proteins CD73, CD39, A2AR, and A2BR, respectively, in human cancers. Data were retrieved from the TCGA database (https://portal.gdc.cancer.gov/). LUAD: Lung adenocarcinoma; LUSC: Lung squamous cell carcinoma; PRAD: Prostate adenocarcinoma; HNSC: Head and neck squamous cell; KIRC: Kidney renal clear cell carcinoma; UCEC: Uterine corpus endometrial carcinoma; PCPG: Pheochromocytoma and paraganglioma; LIHC: Liver hepatocellular carcinoma; COAD: Colon adenocarcinoma; READ: Rectum adenocarcinoma; PAAD: Pancreatic adenocarcinoma; BLCA: Bladder urothelial carcinoma; CESC: Cervical squamous cell carcinoma; CHOL: Cholangiocarcinoma; ESCA: Esophageal carcinoma; KICH: Kidney renal clear cell carcinoma; KIRP: Kidney renal papillary cell carcinoma; STAD: Stomach adenocarcinoma; THYM: Thymoma; THCA: Thyroid carcinoma; BRCA: Breast invasive carcinoma; GBM: Glioblastoma multiforme

Collectively, these observations highlight the multifaceted role of adenosine in tumor progression. Accordingly, we performed a pan-cancer analysis of mutations and DNA methylation alterations in key adenosine-related enzymes and receptors to examine the mechanisms underlying dysregulation of the adenosine pathway in cancer. The analysis revealed significant heterogeneity in the genomic and epigenetic profiles of adenosinergic pathway components (Fig. 2). Mutation frequencies of these genes in cancer are generally low, with the exception of skin cutaneous melanoma (SKCM) and colon adenocarcinoma (COAD), which show a slightly higher prevalence of CD39 mutations. This suggests that while genetic alterations in this pathway are rare, they may still play a role in adenosine dysregulation in specific cancers (Fig. 2A). Therefore, the dysregulation of adenosine-related molecules in tumors is primarily driven by epigenetic modifications and transcriptional regulation rather than genetic mutations. For instance, hypoxia-inducible factor 1α (HIF1α), a central regulator of hypoxic responses, significantly upregulates the expression of CD39, CD73, and A2BR through transcriptional activation. As a result, these molecules are often overexpressed in various cancers and are frequently linked to poor patient prognosis [68]. A2AR gene transcription is uniquely regulated by NF-κB (nuclear factor‑κB), underscoring the complexity of adenosine pathway regulation in cancer [69]. An inverse correlation between DNA methylation and the expression of key adenosinergic pathway genes was observed, suggesting that hypomethylation is linked to their upregulation (Fig. 2B).

In particular, bladder urothelial carcinoma, thymoma, SKCM, pancreatic adenocarcinoma, and COAD exhibit pronounced hypomethylation of adenosine pathway components, impacting both adenosine-generating enzymes and related receptors. These cancers may be favorably responsive to adenosine-targeted therapies. In other cancers, hypomethylation tends to affect only specific ectonucleotidases or receptors, rather than the entire pathway. For example, gene-expression analysis in esophageal carcinoma reveals a CD73-A2BR axis that appears to drive tumor progression (Fig. 2B). Based on these genomic and epigenetic findings, the distribution of adenosine signaling molecules within the TME was also examined.

## The complexed effects of adenosine in the tumor microenvironment

**Fig. 3:**
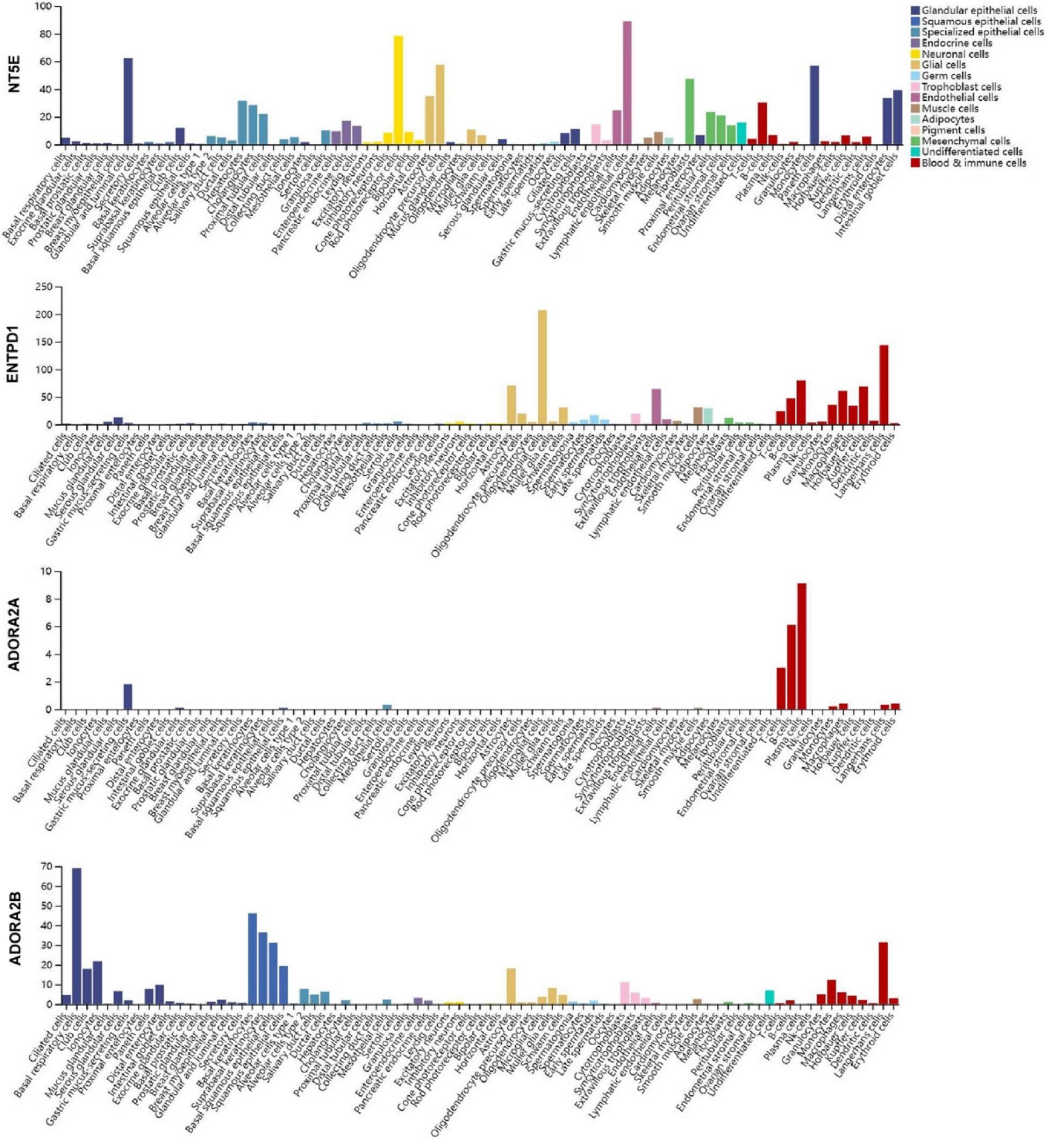
Single cell-type specific landscape of CD73, CD39, A2AR, and A2BR in the adenosine signaling pathway. Single-cell expression profiles of NT5E, ENTPD1, ADORA2A, and ADORA2B, which encode the proteins CD73, CD39, A2AR, and A2BR, respectively, in human cancers and normal tissues, were retrieved from the Human Protein Atlas (HPA) database (https://www.proteinatlas.org/)

To further characterize the distribution of adenosine signaling molecules in single-cell resolution, single-cell RNA-seq data from various tissues were analyzed to define cell-type-specific expression patterns (Fig. 3). CD73 and CD39 are widely expressed not only by cancer cells but also by infiltrating immune and stromal cells, particularly cancer-associated fibroblasts (CAFs). In contrast, A2AR shows cell-type-specific enrichment, primarily in immune cells, while A2BR is predominantly expressed in myeloid cells. Thus, adenosine signaling within the TME involves complex interactions among tumor, immune, and stromal cells, rather than a straightforward tumor-immune exchange. Building on these findings, the role of adenosine in reshaping the immunosuppressive TME was further examined.

### Effects of eADO on immune cells

#### Effects on T lymphocytes

T cells, key effectors of the adaptive immune system, recognize specific antigens to provide long-term defense against pathogens [70–74]. Activation of A2AR by eADO elevates intracellular cAMP levels, which, through PKA activation, impair TCR-mediated signaling and IL-2 receptor-mediated signal transduction [75, 76]. This disruption affects critical T cell functions, including proliferation, motility, cytotoxicity, and cytokine secretion [75, 77–79]. While T cell proliferation is only marginally affected by A2AR activation [80], effector CD8+ T cell cytotoxicity and cytokine production are significantly diminished, indicating that T cells retain proliferative capacity but lose tumor cell elimination ability. Additionally, A2AR-mediated adenosine signaling regulates the PKA/mTORC1 pathway, which is crucial for the metabolic fitness of CD8+ T cells [76]. PKA activation increases intracellular K^+^ levels by inhibiting K^+^ efflux channels [75], further dampening T cell activity. A2AR signaling also contributes to T cell anergy and promotes the differentiation of CD4+ T cells into Tregs [81]. Co-culture of CD4+ Foxp3+ Treg cells with A2AR agonists upregulates CTLA-4 expression, enhances immunosuppressive activity, and significantly increases both the number and function of Treg cells [82], thereby amplifying their immunosuppressive effect.

#### Effects on NK cells

Natural killer (NK) cells play a key role in the surveillance and elimination of infected cells and tumors but are also suppressed by adenosine [83–85]. A2AR stimulation suppresses NK cell maturation, proliferation, cytokine release, and cytotoxicity [86, 87]. Specifically, through A2AR activation, adenosine can inhibit the secretion of IFN-γ, TNF, and perforin 1 (PRF1), while also limiting the production of Fas ligand (FASL) and CD56 [86].

#### Effects on dendritic cells

Dendritic cells (DCs), essential antigen-presenting cells, serve as a link between innate and adaptive immunity [88–90]. Increasing evidence suggests that adenosine plays a pivotal role in the differentiation of myeloid DCs from monocyte/macrophages [91]. Adenosine-exposed DCs exhibit enhanced secretion of angiogenic factors and Th2-type cytokines, promoting angiogenesis, immune suppression, and tolerance via A2BR signaling [91, 92]. Functionally, tolerogenic DCs exhibit a reduced capacity for CD8+ T cell priming in vitro [93]. Mechanistically, adenosine/cAMP signaling polarizes DCs toward a tumor-promoting suppressive phenotype via PKA/Epac pathways [92]. Additionally, adenosine induces mixed cytokine production in DCs [91], including elevated levels of IL-10, TGF-β, VEGF, IL-6, IL-8, indoleamine 2,3-dioxygenase (IDO), and cyclooxygenase 2, while suppressing pro-inflammatory cytokines such as IL-12, TNF-α, and co-stimulatory molecules CD80 and CD86 [91, 92]. This cytokine shift further skews the immune response toward a Th2 phenotype.

#### Effects on neutrophils

Neutrophils, the most abundant white blood cells in humans, are central to the innate immune response and acute inflammation [94]. They represent the first line of defense against pathogens such as bacteria, fungi, and protozoa [94, 95]. Adenosine has several effects on neutrophils. Activation of A1R and A3R enhances neutrophil chemotaxis and phagocytosis, while A2AR and A2BR activation suppresses neutrophil activity by inhibiting adhesion and migration across the endothelial barrier [96]. Specifically, A2R activation suppresses neutrophil effector functions, including reactive oxygen species (ROS) generation, degranulation, Fc receptor-mediated phagocytosis, and the secretion of TNF-α and MIP-1α [28, 96, 97]. Additionally, adenosine plays a pivotal role in neutrophil extracellular trap (NET) formation, where neutrophils release web-like structures composed of DNA, histones, and granular proteins [96–98]. A1R and A3R signaling promote NET formation via ROS and peptidyl arginine deiminase-dependent pathways [96], whereas A2AR activation inhibits NET formation through the cAMP/PKA axis [97].

#### Effects on macrophages

Macrophages can be categorized into two subtypes based on the cytokine environment present during activation [99–102]. When stimulated by Th1 cytokines such as TLR, TNF-α, IFN-γ, and CSF2, macrophages differentiate into an ‘M1-like’ phenotype, which exhibits anti-tumoral activity and secretes pro-inflammatory cytokines like IL-6, IL-12, and IFN-γ [103, 104]. Conversely, exposure to Th2 cytokines like IL-4 and IL-13 drives macrophages toward an ‘M2-like’ phenotype, characterized by increased production of immunosuppressive factors such as IL-10, VEGF, and arginase 1, along with reduced levels of TLR, TNF-α, IFN-γ, and IL-12 [100, 105]. Adenosine influences macrophage polarization, promoting a tolerogenic and pro-tumor ‘M2-like’ phenotype via A2AR and A2BR signaling [105]. Pro-tumor M2 macrophages express elevated levels of A2AR, the primary target of adenosine signaling [106]. Furthermore, adenosine can impair macrophage antibody-dependent cellular phagocytosis (ADCP) by acting as a "don't eat me" signal, hindering the phagocytic process [107].

#### Effects on MDSCs

Myeloid-derived suppressor cells (MDSCs) are a heterogeneous population of immature myeloid cells derived from the bone marrow, playing a key role in regulating immune responses and promoting immune tolerance [108–115]. A2AR activation in MDSCs stimulates IL-10 secretion [116], while A2BR signaling enhances VEGF production through a STAT3-dependent pathway, promoting angiogenesis [117]. Interestingly, A2BR stimulation also activates the cAMP/PKA signaling pathway in MDSCs, resulting in increased CREB phosphorylation, further modulating their immune-suppressive function [118].

### Effects of eADO on tumor cells

Previous studies have highlighted the significant overexpression of CD73 and CD39 in various human tumors, including lung cancer, ovarian cancer, kidney cancer, melanoma, and head and neck squamous cell carcinoma [119–123]. Aberration in the expression of adenosine-generating enzymes in the tumor microenvironment is well-known to promote tumor growth, metastasis, metabolic fitness, and immune evasion in a tumor-autonomous manner [119, 121, 123–125]. Notably, CD73 expression contributes to tumor progression beyond its nucleotidase activity. Preclinical studies suggest that CD73 contributes to the epithelial-mesenchymal transition (EMT), a process critical for metastasis. CD73 functions as a receptor for extracellular matrix proteins, facilitating cell adhesion and migration [126–128]. An in silico analysis of RNA sequencing data from various cancers, particularly prostate adenocarcinoma, revealed a significant correlation between the EMT score and the expression of CD73 and CD39 [128]. In hepatocellular carcinoma (HCC) cells, CD73 promotes progression and EMT through activation of the PI3K-AKT signaling pathway via the Rap1/P110β cascade [129]. CD73 has also been shown to exert pro-stemness activity, enhancing the transcription and stability of SOX9 via the AKT-c-Myc axis [130]. In models of pancreatic ductal adenocarcinoma (PDAC), CD73 competes with Snail for binding to TRIM21, preventing Snail degradation by the proteasome, thereby further promoting EMT and metastasis [124]. Additionally, CD73 has been identified as an independent poor prognostic biomarker for both overall survival (OS) and therapeutic resistance in PDAC and HCC [124, 129, 131].

The expression of A2AR and A2BR is significantly elevated in several solid tumors, including HCC, bladder urothelial carcinoma, and gastric adenocarcinoma [120, 132]. In these cancers, the CD73/adenosine/A2AR pathway transcriptionally upregulates CCL5 through the p38-STAT1 axis, which recruits Tregs to pancreatic tumors and promotes an immunosuppressive microenvironment via tumor-autonomous and autocrine mechanisms [121]. In triple-negative breast cancer (TNBC) models, A2BR signaling activates the p38 MAPK pathway, promoting the nuclear translocation of chromatin remodeling factor SMARCD3 [133]. This pathway further recruits demethylase KDM6A and acetyltransferase p300 to the pluripotency factors *NANOG, SOX2*, and *KLF4,* enhancing breast cancer stemness [133]. Therefore, A2BR signaling is essential for both the induction and maintenance of breast cancer stemness, particularly under the hypoxic conditions typically present in the TME [134, 135].

### Effects of eADO on tumor stromal cells

Stromal cells in the TME are key drivers of tumor progression through the adenosine pathway. CAFs, the predominant non-hematopoietic stromal cells, contribute to tumor progression, chemoresistance, metastasis, and cancer stem cell maintenance [136–139]. CAFs promote tumor progression by secreting immunomodulatory molecules, interacting with immune cells, and remodeling the extracellular matrix [136–138, 140, 141], thus collaborating with other TME components to sustain tumor growth [142].

**Fig. 4:**
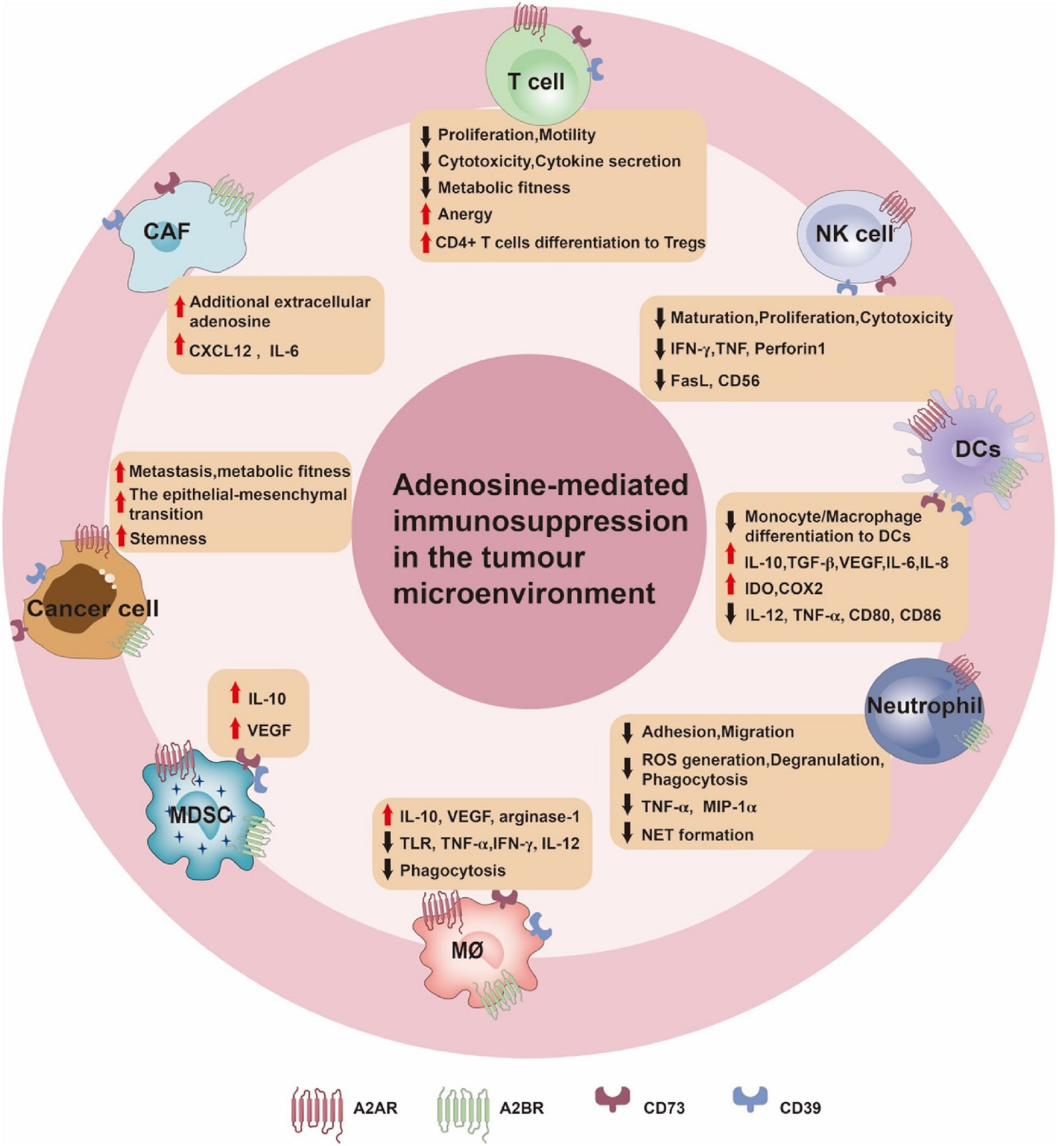
Immunosuppressive effects of adenosine within the tumor microenvironment. The tumor microenvironment is composed of a diverse array of immune and non-immune cells, each exhibiting distinct expression profiles of functional adenosine receptors and adenosine-generating enzymes, mainly including A2AR, A2BR, CD39, and CD73. Adenosine facilitates tumor immune evasion by impairing protective immune components such as DCs, NK cells, T cells, and neutrophils, while simultaneously promoting the activity of immunosuppressive cells, including Tregs, M2 macrophages, and MDSCs. Targeting the various adenosinergic pathways may effectively reverse the adenosine-mediated immunosuppressive microenvironment. DCs: Dendritic cells; NK cell: Natural killer cell; Treg cells: Regulatory T cells; MDSC: Myeloid-derived suppressor cell; Mø: Macrophage; CAF: Cancer-associated fibroblast

CD39 and CD73, highly expressed on CAFs, are found in a variety of human tumors, including breast, colorectal, ovarian, and pancreatic cancers, where they contribute to the generation of additional immunosuppressive adenosine within the TME [86, 143]. In patients with colorectal cancer (CRC), elevated CD73 levels correlate with increased CAF abundance, and CD73 expression on CAFs is essential for maintaining the immunosuppressive environment [139]. CAFs further amplify CD73 expression via an A2BR-mediated feed-forward loop triggered by tumor cell death, resulting in additional adenosine production [139]. A2BR signaling on CAFs also enhances CXCL12 secretion, which recruits Treg cells to the tumor and promotes T lymphocyte differentiation into CD25^high^Foxp3^high^ subsets [144, 145], potentially fostering pro-tumor effects both autocrine and paracrine. In breast cancer, a positive feedback loop involving CAFs and CD73+ γδ Tregs stimulates IL-6 secretion by CAFs via the adenosine/A2BR/p38MAPK signaling pathway, further contributing to immunosuppression [146]. Additionally, bidirectional interactions between T cells and CAFs in non-small cell lung cancer (NSCLC) promote components of the immunosuppressive CD39/CD73 adenosine pathway [147]. A summary of adenosine-induced effects across these cell types is provided (Fig. 4).

## Current therapeutic strategies for targeting the eADO pathway

**Fig. 5:**
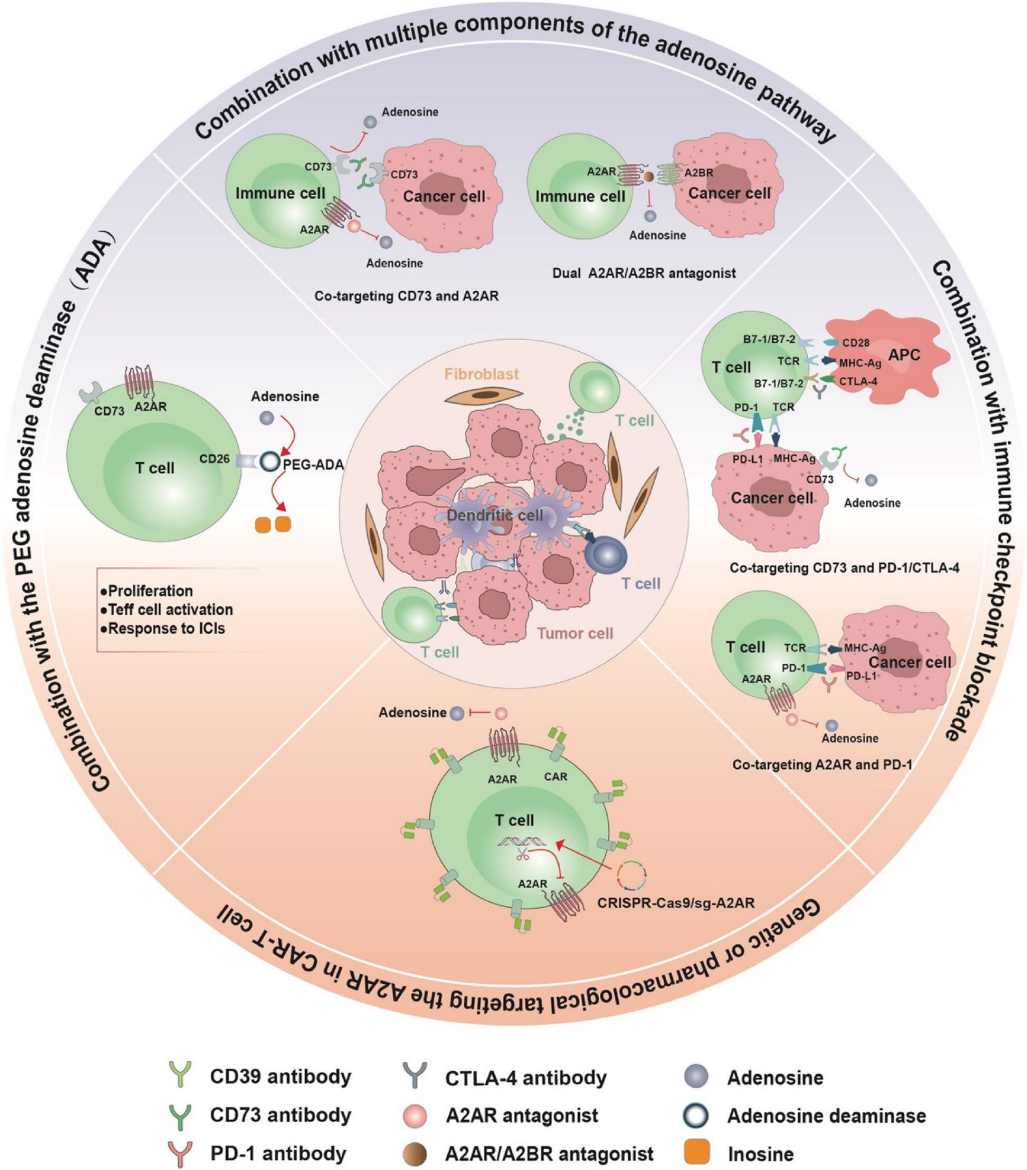
Potential for combining inhibition of the adenosinergic pathway and other cancer immunotherapies. Co-targeting key components of the adenosinergic pathway, such as A2AR, A2BR, CD39, and CD73, offers synergistic therapeutic potential by modulating both tumor and immune cells. Furthermore, adenosinergic pathway inhibitors may be effectively combined with other cancer immunotherapies, such as immune checkpoint blockade (ICB) and adoptive cellular therapy (ACT), to improve treatment outcomes across various cancers. This strategy is under active investigation and will be further evaluated in large-scale clinical trials. Additionally, targeting adenosine deaminase (e.g., PEG-ADA), which promotes inosine generation, remains a potential approach, though it has yet to be tested

As a key metabolite in the TME, adenosine exerts potent immunosuppressive effects and facilitates tumor progression. Current clinical trials targeting adenosine pathway components aim to enhance antitumor responses, focusing on three main strategies: 1) inhibiting adenosine production, 2) blocking adenosine receptor binding, and 3) combining adenosine pathway inhibitors with other cancer immunotherapies. A comprehensive overview of ongoing and investigational clinical trials targeting the adenosine pathway is presented in Table 1. To enhance clarity, Table 2 provides a separate summary of completed clinical trials, highlighting both adverse events (AEs) and clinical outcomes. This summary reviews ongoing preclinical and clinical studies in this field (Fig. 5).

**Table 1:** Ongoing clinical trials of eADO pathway-targeting drugs in patients with malignancies

Target	Drug	Type	Study phase	Number of patients	Cancer type	Status	Combination partner	AEs	Efficacy				NCT number
									**ORR**	**DCR**	**Median PFS**	**Median OS**	
CD39	ES002023	antibody	I	60	LA/M Solid Tumors	Active, not recruiting	N/A	N/A	N/A	N/A	N/A	N/A	NCT05075564
	TTX-030	antibody	II	194	1L mPDAC	Active, not recruiting	•Budigalimab (anti-PD-1 mAb) •Gemcitabine + Nab-Paclitaxel	N/A	N/A	N/A	N/A	N/A	NCT06119217
	ES014	antibody	I	120	LA/M Solid Tumors	Recruiting	N/A	N/A	N/A	N/A	N/A	N/A	NCT05717348
	JS019	antibody	I	72	Advanced Solid Tumors	Recruiting	N/A	N/A	N/A	N/A	N/A	N/A	NCT05508373
CD73	PM1015	antibody	I	20	Advanced Solid Tumors	Recruiting	N/A	N/A	N/A	N/A	N/A	N/A	NCT05950815
	GS-1423 (Dalutraf-usp Alfa)	antibody	I	22	Advanced Solid Tumors	Terminated (No safety concerns were observed)	mFOLFOX6	Grade 3-4 AEs in 42.9% (9/21); common grade 1-2 AEs: fatigue (47.6%), nausea (33.3%), diarrhea (28.6%), and vomiting (28.6%)	4.8% (1 PR/17 pts)	38.1% (1 PR + 7 SD/17 pts)	N/A	N/A	NCT03954704
	JAB-BX102	antibody	I/II	62	Advanced Solid Tumors	Recruiting	Pembrolizumab (anti-PD-1 mAb)	N/A	N/A	N/A	N/A	N/A	NCT05174585
	IPH5301	antibody	I	27	Endometrial Cancer, Metastatic Breast Cancer, Metastatic Gastric Cancer, Metastatic Lung Cancer, Metastatic Ovary Cancer, Metastatic Pancreatic Cancer, Oesophageal Cancer	Recruiting	Chemotherapy and Trastuzumab (anti-HER-2 mAb)	N/A	8.3% (1 PR/12 pts)	41.7% (1 PR + 4 SD/12 pts)	N/A	N/A	NCT05143970 (https://oncologypro.esmo.org/meeting-resources/esmo-congress-2024/a-first-in-human-fih-phase-i-study-of-iph5301-an-anti-cd73-monoclonal-antibody-mab-in-patients-with-advanced-solid-tumors-ast-chances-nct)
	HB0045	antibody	I/II	71	Advanced Solid Tumors	Recruiting	N/A	N/A	N/A	N/A	N/A	N/A	NCT06056323
	PT199	antibody	I/II	40	NSCLC, PDAC	Recruiting	Chemotherapy or Tislelizumab (anti-PD-1 mAb)	N/A	N/A	N/A	N/A	N/A	NCT05431270
	TJ004309	antibody	I	36	Advanced Solid Tumors	Active, not recruiting	Atezolizumab (anti-PD-L1 mAb)	First-dose infusion-related reactions were observed in 65% of patients; most common AEs were grade 1-2 chills/rigors, nausea, and vomiting	23% (1 CR, 2 PR/13 pts)	46% (3 CR/PR + 3 SD/13 pts)	N/A	N/A	NCT03835949 (https://ascopubs.org/doi/abs/10.1200/JCO.2021.39.15_suppl.2511)
			I/II	376	Advanced Solid Tumors	Active, not recruiting	Toripalimab (anti-PD-1 mAb)	Three grade 3-4 AEs (decreased lymphocyte count and transient QT prolongation); most common AEs were grade 1-2 chills (47.8%), vomiting (46.7%), pyrexia (40.2%), diarrhea (32.6%), nausea (19.6%), pruritus (14.1%) and rash (13%)	12.5% (6 PR/48 pts)	56.4% (6 PR + 21 SD/48 pts)	N/A	N/A	NCT04322006 (https://ascopubs.org/doi/pdf/10.1200/JCO.2022.40.16_suppl.e21123)
	S095024	antibody	I/II	176	NSCLC	Recruiting	•S095018 (anti-TIM3 mAb) •S095029 (anti-NKG2A mAb)	N/A	N/A	N/A	N/A	N/A	NCT06162572
	AB680	antibody	I	195	Advanced Pancreatic Cancer	Recruiting	Zimberelimab (anti-PD-1 mAb) + Nab-paclitaxel + Gemcitabine	Anemia (14%, 2/13) was the most common grade 3-4 AEs; most frequent AEs were grade 1-2 fatigue (43%), anemia (29%), and neutrophil count decrease (29%)	33.3% (3 PR/9 pts)	88.9% (3 PR + 5 SD/9 pts)	N/A	N/A	NCT04104672 (https://ascopubs.org/doi/abs/10.1200/JCO.2021.39.3_suppl.404)
	NZV930	antibody	I	127	MSS, mCRPC, NSCLC, Ovarian Cancer, PDAC, Renal Cell Carcinoma, TNBC	Terminated (Termination was not safety related)	Spartalizumab (anti-PD-1 mAb) ± NIR178 (small-molecule A2AR antagonist)	Four DLTs: grade3-4 headache; most frequent AEs were headache (67%), nausea and vomiting (32% each), and pyrexia (30%)	N/A	11% (12 SD/105 pts)	N/A	N/A	NCT03549000 (https://aacrjournals.org/cancerres/article/82/12_Supplement/CT503/704432/Abstract-CT503-A-phase-I-Ib-study-of-the-safety)
A2AR	Ciforadenant	antagonist	I/II	24	Renal Cell Carcinoma	Recruiting	Ipilimumab (anti-CTLA-4 mAb) + Nivolumab (anti-PD-1 mAb)	N/A	N/A	N/A	N/A	N/A	NCT05501054
	TT-10 (PORT-6)	antagonist	I/II	90	CRPC, HNSCC, NSCLC, Renal Cell Cancer	Recruiting	TT-4 (small-molecule A2BR antagonist)	All AEs were grade 1-2; most common AEs were fatigue (29%), nausea (29%), and vomiting (14%); no DLTs were observed	N/A	N/A	N/A	N/A	NCT04969315 (https://doi.org/10.1200/JCO.2024.42.16_suppl.e14681)
	Inupadenant (EOS100850)	antagonist	II	186	LA/M NSCLC	Recruiting	Carboplatin + Pemetrexed	No treatment-related deaths; AEs consistent with platinum-doublet chemotherapy	63.9% (Overall); 53.3% (Inupade-nant 40 mg); 73.3% (Inupade-nant 80 mg)	N/A	5.6 months (Inupade-nant 40 mg); ≥ 6-month follow-up not reached (Inupade-nant 80 mg)	N/A	NCT05403385 (https://www.sitcancer.org/blogs/thomas-martin/2024/12/16/esmo-io-meeting-2024-dec-79)
A2BR	PBF-1129	antagonist	I	18	LA/M NSCLC	Active, not recruiting	N/A	No DLTs were observed; three grade 3-4 AEs (lymphocytopenia, hyponatremia, hypertension, and encephalopathy); most common AEs were lymphocytopenia (38%), vomiting (38%), anorexia (29%), and fatigue (29%)	N/A	16.7% (3 SD/18 pts)	1.5 months (95% CI: 1.0-1.9)	4.6 months (95% CI: 2.1-5.2)	NCT03274479 (https://jitc.bmj.com/content/10/Suppl_2/A612)
	TT-702	antagonist	I/II	188	Advanced Solid Tumors	Recruiting	Darolutamide	N/A	N/A	N/A	N/A	N/A	NCT05272709
A2AR and A2BR	M1069	antagonist	I	15	LA/M Unresectable Solid Tumors	Terminated (The study was not terminated due to safety)	N/A	N/A	N/A	N/A	N/A	N/A	NCT05198349

*AE* adverse event, *AR* androgen receptor, *bsAb* bispecific antibody, *CR* complete response, *DCR* disease control rate, *DLT* dose-limiting toxicity, *eADO* extracellular adenosine, *HNSCC* head and neck squamous cell carcinoma, *LA/M* locally advanced or metastatic, *mAb* monoclonal antibody, *mCRPC* metastatic castration-resistant prostate cancer, *MSS* microsatellite stable, *N/A* not applicable, *NSCLC* non-small cell lung cancer, *ORR* objective response rate, *OS* overall survival, *PDAC* pancreatic ductal adenocarcinoma, *PFS* progression-free survival, *PR* partial response, *pts* patients, *SD* stable disease, *TNBC* triple-negative breast cancer, 1L mPDAC first-line treatment of metastatic pancreatic ductal adenocarcinoma

**Table 2:** Summary of completed clinical trials targeting the adenosine pathway in cancer immunotherapy

Target	Drug	Type	Study phase	Number of patients	Cancer type	Combination partner	AEs	Efficacy				NCT number
								**ORR**	**DCR**	**Median** **PFS** **(months)**	**Median OS** **(months)**	
CD39	TTX-030	antibody	I	56	Solid Tumors, Lymphoma	•Pembrolizumab (anti-PD-1 mAb) •Gemcitabine + Nab-paclitaxel	N/A	N/A	N/A	N/A	N/A	NCT03884556
		antibody	I	185	Advanced Solid Tumors	•Budigalimab/Pembrolizumab (anti-PD-1 mAb) •FOLFOX •Gemcitabine + Nab-Paclitaxel	The most common AEs were nausea (52%), neutrophil count decreased (39%), decreased appetite (30%), diarrhea (25%), and fatigue (23%). The most common grade ≥ 3 AEs were neutrophil count decreased (27%), febrile neutropenia (5%), hypokalemia (5%)	61% (2 CR, 21 PR/38 pts)	92% (23 CR/PR + 12 SD/38 pts)	N/A	N/A	NCT04306900 (https://www.abstractsonline.com/pp8/#!/10517/presentation/20157)
	IPH5201	antibody	I	57	Advanced Solid Tumors	Durvalumab (anti-PD-L1 mAb) ± Oleclumab (anti-CD73 mAb)	N/A	N/A	38.6% (22/57)	1.4 (0-–15.2)	8.2 (1.0-22.1)	NCT04261075 (https://www.esmoiotech.org/article/S2590-0188(22)00231-3/fulltext)
	SRF617	antibody	I	85	Advanced Solid Tumors	•Pembrolizumab (anti-PD-1 mAb) •Gemcitabine + Albumin-bound paclitaxel	No DLTs were observed; the most common AEs in monotherapy were fatigue (35%), nausea (22%), and constipation (19%)	N/A	N/A	N/A	N/A	NCT04336098 (https://www.annalsofoncology.org/article/S0923-7534(21)04690-1/fulltext)
CD73	LY3475070	antibody	I	52	Advanced Solid Tumors	Pembrolizumab (Pembro, anti-PD-1 mAb)	Most frequent AEs were anaemia, diarrhoea, nausea, chills and fatigue	N/A	50% (150 QD) 33.3% (300 QD) 16.7% (300 BID) 0% (600 QD) 66.7% (150 QD + Pembro) 27.3% (150 BID + Pembro) 0% (300 QD + Pembro) 35.3% (300 BID + Pembro)	2.71 (0.03-3.42, 150 QD) 1.91 (0.03-4.8, 300 QD) 0.89 (0.03-2.14, 300 BID) 1.33 (0.03-–2.04, 600 QD) 2.00 (0.03-–2.04, 150 QD + Pembro) 0.53 (0.03-3.52, 150 BID + Pembro) 0.03 (0.03-–0.03, 300 QD + Pembro) 0.03 (0.03-2.5, 300 BID + Pembro)	N/A	NCT04148937
	AK119	antibody	I	23	Advanced or Metastatic Solid Tumors	Candonilimab (anti-PD-1/CTLA-4 bsAb)	N/A	N/A	N/A	N/A	N/A	NCT04572152
	IBI325	antibody	I	48	Advanced Solid Tumors	Sintilimab (anti-PD-1 mAb)	N/A	N/A	N/A	N/A	N/A	NCT05119998
	Sym024 (S095024)	antibody	I	48	Advanced Solid Tumors	Sym021 (anti-PD-1 mAb)	The most frequent AEs (≥ 15%) were fatigue, nausea, diarrhea, dyspnea, and vomiting	N/A	N/A	N/A	N/A	NCT04672434 (https://aacrjournals.org/cancerres/article/84/6_Supplement/3737/739522/Abstract-3737-Molecular-and-early-clinical)
	MEDI9447 (Oleclumab)	antibody	I	192	Colorectal Cancer, PDAC, NSCLC	Durvalumab (anti-PD-L1 mAb)	Grade 3-4 AEs reported in the monotherapy cohort were ascites (12%), hyperglycemia (7%), acute kidney injury, anemia, hyponatremia, and hypotension (each 5%), and in the combination therapy cohort were alanine aminotransferase increased, aspartate aminotransferase increased, blood bilirubin increased, and pulmonary embolism (each 8%); most frequent AEs were fatigue (15%), diarrhea (9%), and rash (7%)	2.4% (Colorectal Cancer); 4.8% (PDAC); 9.5% (NSCLC)	N/A	6-month PFS rate (%): 5.4% (Colorectal Cancer); 13.2% (PDAC); 16.0% (NSCLC)	N/A	NCT02503774 (PMID: 37016126)
	ORIC-533	antibody	I	31	Relapsed or Refractory Multiple Myeloma	N/A	No DLTs were observed; no grade 4 AEs were reported; fatigue was the most frequent AE	N/A	N/A	N/A	N/A	NCT05227144 (https://doi.org/10.1182/blood-2023–173730)
	CPI-006	antibody	I	117	NSCLC, Renal Cell Cancer, Colorectal Cancer, TNBC, Cervical Cancer, Ovarian Cancer, Pancreatic Cancer, Endometrial Cancer, Sarcoma, SCCHN, Bladder Cancer, mCRPC, Non-hodgkin Lymphoma	•Ciforadenant (small-molecule A2AR antagonist) •Pembrolizumab (anti-PD-1 mAb)	No DLTs with monotherapy or combination therapy were observed	Not formally reported; tumor regression in 1 prostate cancer patient	N/A	N/A	N/A	NCT03454451 (https://ascopubs.org/doi/abs/10.1200/JCO.2019.37.15_suppl.2505)
A2AR	NIR178 (PBF-509)	antagonist	I	92	NSCLC	PDR001 (anti-PD-1 mAb)	One DLT: grade 3 nausea; most frequent AEs were nausea (67%), fatigue (63%), dyspnea (46%), vomiting (33%)	11.8% (1 CR, 1 PR/17 pts)	47.1% (8/17)	N/A	N/A	NCT02403193 (https://ascopubs.org/doi/abs/10.1200/JCO.2018.36.15_suppl.9089)
	Ciforadenant	antagonist	I	502	Renal Cell Cancer, mCRPC	Atezolizumab (Atezo, anti-PD-L1 mAb)	Grade 3-4 AEs in 12.1% (4/33) with monotherapy (decreased appetite, anemia, arthralgia, edema peripheral) and grade 3-4 AEs in 14.3% (5/35) with combination (nausea, arthralgia, hypophosphatemia, abdominal pain, AST increased); most frequent AEs were fatigue, pruritus, decreased appetite and nausea	3% (mono); 11% (+ Atezo)	17.0% (6-month, mono); 39.0% (6-month, + Atezo)	4.1 months (mono); 5.8 months (+ Atezo)	69% (25-month, mono); 90% (25-month, + Atezo)	NCT02655822 (PMID: 31732494)
	AZD4635	antagonist	I	313	NSCLC, mCRPC, Colorectal Carcinoma	Durvalumab (Durva, anti-PD-L1 mAb)	Two DLTs: grade 2 fatigue and grade 2 nausea (in AZD4635 + durvalumab cohort); most frequent AEs were nausea, fatigue, vomiting, decreased appetite, dizziness, and diarrhea	5.1% (2/39, mono); 16.2% (6/37, + Durva)	N/A	4.8 months (high adenosine sigature); 2.0 months (low adenosine sigature)	N/A	NCT02740985 (PMID: 36044531)
A2AR and A2BR	Etrumadenent (AB928)	antagonist	I/II	173	mCRPC	•Zimberelimab (anti-PD-1 mAb) •Quemliclustat (anti-CD73 mAb) •Enzalutamide (small-molecule AR antagonist) •Docetaxel •Sacituzumab govitecan	Grade 3-4 AEs in 53% (9/17); most frequent AEs were lymphocyte count decreased, neutrophil count decreased, hyponatremia and alopecia	38% (3/8)	43% (6/14)	N/A	N/A	NCT04381832 (https://ascopubs.org/doi/abs/10.1200/JCO.2021.39.15_suppl.5039)

*AE* adverse event, *DLT* dose-limiting toxicity, *mAb* monoclonal antibody, *N/A* not applicable, *PDAC* pancreatic ductal adenocarcinoma, *NSCLC* non-small cell lung cancer, *TNBC* triple-negative breast cancer, *SCCHN* squamous cell carcinoma of the head and neck, *mCRPC* metastatic castration-resistant prostate cancer, *pts* patients, *DCR* disease control rate, *ORR* objective response rate, *PFS* progression-free survival, *OS* overall survival, *CR* complete response, *PR* partial response, *SD* stable disease, *QD* once daily (quaque die), *BID* twice daily (bis in die). All listed studies were completed, clinical efficacy and adverse events are summarized from reported results

### Blockade of adenosine generation

#### Blockade of CD73

Four distinct monoclonal antibodies (mAbs) targeting CD73 are currently being investigated in clinical trials: oleclumab (MEDI9447), BMS-986179, CPI-006, and NZV930. Oleclumab, a human IgG1 mAb, specifically blocks CD73 in mouse models, triggering immunomodulatory effects, including increased CD8+ T cell infiltration and macrophage activation [148]. Preliminary data indicate that oleclumab, either alone or in combination with anti-PD-L1, exhibits a tolerable safety profile and promising antitumor efficacy in advanced CRC, PDAC, and EGFR-mutant NSCLC (NCT02503774) [149–151]. A Phase 1/2a trial initiated in 2016 is evaluating the anti-tumor efficacy of BMS-986179, both as monotherapy and in combination with anti-PD-1 (nivolumab), across various solid tumors (NCT02754141). Preliminary results indicate that BMS-986179 combined with nivolumab shares a safety profile comparable to nivolumab alone in the treatment of advanced solid tumors [152]. CPI-006, a humanized IgG1 FcR-binding-deficient antibody, has been shown to rapidly redistribute lymphocytes and increase the number of TH effector/memory cells [153].

These anti-CD73 mAbs exert antitumor effects primarily by inhibiting CD73 activity or promoting its internalization. Given CD73's abundant expression in non-malignant tissues, most anti-CD73 mAbs are engineered to block Fc receptor involvement, minimizing immune-mediated cytotoxicity toward non-malignant cells [148]. In addition to anti-CD73 mAbs, small molecule inhibitors of CD73, such as AB680, ORIC-533 [154], and LY3475070 [155], are also being explored. Although clinical data remain limited, these small molecules appear well tolerated and hold potential as promising tools for further research and development [156, 157].

#### Blockade of CD39

In contrast to CD73 targeting, blocking CD39 activity with therapeutic antibodies offers a dual benefit: reducing the production of immunosuppressive eADO and increasing the immunostimulatory molecule eATP. ATP, released into the extracellular space upon cellular stress, cell death, or inflammation, functions as a "natural adjuvant" with proinflammatory effects, including the activation of P2X7R [23, 25, 158, 159]. Pharmacological blockade of CD39 promotes macrophage engulfment of antibody-coated tumor cells in a P2X7R-dependent manner [160]. P2X7R activation leads to an intracellular influx of K^+^, a key trigger for NLRP3 inflammasome activation [161]. The inflammasome subsequently controls the maturation and release of cytokines such as interleukin-1β (IL-1β) and interleukin-18 (IL-18), both of which are critical for immune responses [162–164]. Furthermore, CD39 blockade with therapeutic antibodies activates the eATP-P2X7R-inflammasome-IL-18 axis, potentially reducing intratumor macrophage populations and enhancing T cell effector function, providing a therapeutic advantage beyond merely decreasing adenosine production [165]. Vignali et al. [166] demonstrated that CD39 is crucial for the function of CD8+ Tex cells, which exhibit suppressive potential comparable to CD4+ Foxp3+ Treg cells. Therefore, CD39 serves as a multifunctional target for cancer immunotherapy, due to its central role in ATP degradation and its widespread expression across various cell types in the TME.

To date, five anti-CD39 mAbs are in clinical trials, either as monotherapies or in combination with other therapeutic strategies, including TTX-030 (NCT03884556), SRF617 (NCT04336098), IPH5201 (NCT04261075), ES002023 (NCT05075564), ES014 (NCT05717348) and JS019 (NCT05508373). Preliminary data suggest that anti-CD39 mAbs enhance antitumor effects and show significant synergy when combined with PD-1/PD-L1 inhibitors.

### Blockade of adenosine receptors

#### Blockade of A2AR

A2AR antagonists were initially developed as neurological agents for clinical treatment, suggesting superior penetration properties. Currently, A2AR antagonists are undergoing early-phase clinical trials to assess their antitumor activity and clinical safety. These include AZD4635, taminadenant (NIR178/PBF-509), ciforadenant (CPI-444), EOS100850, and two dual A2A and A2B antagonists (AB928 and M1069). Preclinical studies indicate that NECA, a stable adenosine analog, inhibits antigen presentation and T-cell co-stimulation in CD103+ DCs, effects that AZD4635 treatment can reverse [167, 168]. Antigen presentation by DCs is crucial for priming and expanding antigen-specific T cells. Blockade of A2AR with AZD4635 has been demonstrated to increase intratumoral CD8+ T cells and DCs, thereby reducing tumor burden and enhancing antitumor immunity [167, 168]. The antitumor activity of AZD4635, both as a monotherapy and in combination therapies, has been investigated in a multicenter clinical trial involving 313 patients with advanced solid malignancies (NCT02740985). Preclinical research has also shown that PBF-509, a novel A2AR antagonist, significantly boosts the immune response of tumor-infiltrating lymphocytes and reduces tumor metastasis, either alone or in combination with anti-PD-1/PD-L1 [169]. A Phase I/Ib dose-escalation study confirmed that taminadenant (PBF509/NIR178) was well-tolerated in patients with advanced NSCLC [170]. Similarly, Fong et al*.* [171] demonstrated that ciforadenant, a small-molecule A2AR antagonist, safely blocks adenosine signaling in patients with RCC. Durable clinical benefits, including enhanced CD8+ T cell infiltration into tumors, confirmed the safety and efficacy of targeting this pathway. This first-in-human trial of an A2AR antagonist in cancer treatment underscores the antitumor potential of ciforadenant (CPI-444) both as a monotherapy and in combination with anti-PD-L1 in patients with refractory RCC. Furthermore, the study revealed that patients with adenosine-regulated gene expression profiles in pretreatment tumor biopsies experienced better therapeutic outcomes, suggesting that biomarkers could guide patient selection for targeted adenosine therapy, thereby optimizing treatment efficacy [172].

#### Blockade of A2BR

In contrast to the low-affinity A2BR, A2AR has garnered more attention in the development of therapeutic agents targeting adenosine receptors. As research into the adenosine pathway in the TME advances, an increasing number of preclinical models have reinforced the dominant role of A2BR in immune system regulation [173–176]. Currently, Phase I clinical trials are evaluating the safety and optimal dosing of PBF-1129, a selective A2B antagonist, in patients with locally advanced or metastatic NSCLC (NCT03274479).

## The rationale of combination therapy

Although inhibiting the adenosine pathway shows therapeutic promise, cancer cells may develop resistance through various adaptive mechanisms. When the canonical CD39-CD73 pathway is inhibited, cancer cells can utilize a non-canonical pathway to generate eADO [34, 38]. In this alternative route, NAD⁺ is converted to AMP via the CD38-CD203a axis, which subsequently produces adenosine through other membrane-bound phosphatases. Additionally, compensatory feedback loops often reduce the efficacy of monotherapy targeting the adenosine pathway. For example, CD73 expression is elevated in A2AR-deficient mice [177], suggesting compensatory mechanisms when a single adenosinergic pathway is inhibited. The interplay between the adenosine pathway and other tumor-promoting factors, such as hypoxia and inflammation, further exacerbates resistance to adenosine antagonists, complicating the therapeutic landscape [178]. Moreover, blockade of adenosine signaling may force tumors to rely on alternative immunosuppressive checkpoints, such as PD-L1, to sustain an immunosuppressive environment [66]. Immune cells may also upregulate adenosine-related receptors, increasing their sensitivity to adenosine-induced inhibition. For instance, human-derived CAR-T cells often overexpress A2AR, rendering them more susceptible to adenosine-induced impairment [179].

Collectively, multiple strategies are being developed to counteract adenosine-mediated resistance. Early-phase clinical trials targeting CD73, CD39, A2AR, and A2BR have shown favorable safety profiles and early signs of efficacy, particularly in combination with immune checkpoint inhibitors (Tables 1 and 2). Co-targeting the adenosine pathway with other cancer immunotherapies, such as immune checkpoint blockade or adoptive T-cell transfer, has demonstrated synergistic antitumor effects in preclinical studies. These advancements highlight the potential of adenosine pathway inhibition as a complementary approach in cancer treatment and provide a strong rationale for ongoing combination trials.

### Simultaneously targeting multiple components of the adenosine pathway

Blocking CD73 using monoclonal antibodies or small molecule inhibitors is the predominant approach for targeting the adenosine pathway to inhibit extracellular adenosine production. In a murine model of PDAC, preclinical research has demonstrated that co-inhibition of CD73 and CD39 yields significantly superior anti-tumor activity [180]. However, as previously noted, CD39-CD73 is not the sole pathway responsible for adenosine production. The CD203a-CD73 axis represents an alternative, CD39-independent adenosinergic loop that may enable cancer cells to bypass CD39-targeted therapies. Cancer cells have been shown to upregulate CD203a in response to CD39 inhibition, maintaining an immunosuppressive TME [43, 181]. Consequently, simultaneous blockade of CD39 and CD73 may not fully inhibit adenosine production under certain conditions, as evidenced by immunohistochemical staining of human tumor specimens [182].

In an alkaline environment, alkaline phosphatases (APs), anchored to the plasma membrane, catalyze the removal of phosphate groups from various substrates, including ATP and ADP [183]. Additionally, PAP exhibits AMPase activity, converting extracellular AMP to adenosine through dephosphorylation [184]. Subsequent studies have shown that PAP expression extends beyond prostate tissue, being present in other malignancies, including breast and colon cancers [185, 186]. Furthermore, preclinical studies have confirmed that PAP interacts synergistically with CD73 in a non-redundant manner to modulate immune function, particularly affecting Treg cell populations in the lymph nodes and thymus [187]. However, it remains unclear whether increased PAP and APs enzymatic activity will be sufficient to compensate for adenosine production following CD73 blockade.

Simultaneous blockade of CD73 and A2AR represents a more viable strategy in scenarios where complete suppression of adenosine synthesis is not feasible. Preclinical models demonstrate that co-targeting CD73 and A2AR outperforms monotherapy in tumor control [188]. In a murine PDAC model, combining an anti-CD73 antibody with an A2AR inhibitor significantly slowed tumor growth and reduced metastatic burden, which correlated with reduced infiltration of M2 macrophages and Treg cells within the TME [106]. Additionally, co-blockade of the CD39/CD73/A2AR adenosinergic pathway resulted in increased IFN-γ secretion and reduced tumor load in a multiple myeloma model, highlighting the potential therapeutic benefit of targeting multiple points within the adenosinergic pathway [189].

AB928, the first clinical-stage small molecule dual A2AR/A2BR antagonist, is currently undergoing evaluation in several Phase 1b clinical trials. Preliminary data indicate that AB928 alleviates adenosine-mediated immunosuppression by blocking A2AR/A2BR-induced signaling and gene expression alterations, thereby suppressing tumor growth in vivo [190–192]. Notably, AB928 appears to be more effective than A2AR-selective antagonists in inhibiting adenosine-induced immunosuppression and gene expression changes in myeloid cells and A2BR-expressing cancer cell lines [191]. This provides a mechanistic rationale for stimulating antitumor immune responses with the dual adenosine receptor antagonist AB928. A Phase 1b/2 trial is currently underway to assess the safety and efficacy of etrumadenant-based treatment combinations in patients with metastatic castrate-resistant prostate cancer (mCRPC) (NCT04381832). In this trial, the most common treatment-related adverse events associated with AB928 were decreased lymphocyte and neutrophil counts [193]. Preclinical data support the manageable safety profile and superior antitumor efficacy of AB928 in patients with mCRPC [193].

### Combination with immune checkpoint blockade

Immune checkpoint blockade (ICB) targeting CTLA-4 and PD-1/PD-L1 has revolutionized cancer care, demonstrating significant success in patients with various advanced cancers [194, 195]. However, the majority of patients do not respond to these therapies [196–200], underscoring the need for further development of agents targeting additional mechanisms of tumor immune evasion. Over recent years, adenosine signaling has been identified as a key metabolic pathway involved in tumor immunity. Combining ICB with adenosine blockade may extend the benefits of immunotherapy to a broader patient population. Preclinical studies have shown that inhibitors of the eADO pathway enhance the antitumor efficacy of ICB. For example, combining CD73 inhibition with either anti-PD-1 or anti-CTLA-4 results in synergistic antitumor activity [148, 201, 202], and A2AR antagonists in combination with ICB also improve efficacy [202–205]. Interestingly, PD-1 inhibition has been shown to increase A2AR expression on tumor-infiltrating CD8+ T lymphocytes, making them more susceptible to A2A-mediated suppression. Moreover, anti-PD-1 or anti-CTLA-4 monotherapy can improve tumor control and delay tumor progression in CD39-knockout mice [206]. Consequently, several combination therapies are currently being tested in clinical trials. Nearly all clinical trials involving eADO pathway inhibitors include a combination arm with ICB or chemotherapy in patients with cancer.

### Combination with adoptive cell immunotherapy

Preclinical studies support the potential of adenosine targeting to enhance the efficacy of adoptive cellular therapy (ACT). ACT utilizes tumor-infiltrating lymphocytes or gene-modified T cells expressing transgenic antigen receptors such as T cell receptors (TCRs) or chimeric antigen receptors (CARs) [207–210]. Although ACT has shown promising results, its efficacy is frequently compromised by adaptive resistance mechanisms in the tumor microenvironment [211, 212]. For example, adoptive T-cell transfer has been shown to increase CD73 expression in melanoma patients, contributing to this resistance [213]. CD39 marks a subset of exhausted human CAR‑T cells, and importantly, many of these cells co‑express CD73 and concurrently mediate immunosuppression via A2AR [214]. Upregulation of A2AR on CAR-T cells can further impair their function via adenosine-mediated immunosuppression. To overcome these challenges, both pharmacological and genetic strategies targeting the adenosine pathway have been explored in preclinical models [179, 205, 215]. The selective A2AR antagonist AB928, for example, protects CAR-T cells from the suppressive effects of adenosine, enhancing cytokine production and proliferation [216]. In parallel, A2AR knockout CAR-T cells using a CRISPR-Cas9 strategy outperform pharmacological blockade of A2AR, showing improved cytokine production, including IFN-γ and TNF [217]. Moreover, engineering CAR-T cells to express enzymes such as adenosine deaminase (ADA) allows the conversion of adenosine to inosine, promoting stemness and enhancing CAR-T functionality [214, 218]. Further preclinical studies suggest that inhibiting CD73 or A2AR can enhance ACT efficacy [179, 215, 219]. A novel A2AR antagonist CPI-444 has been demonstrated to potentiate IFN-γ production in transferred T lymphocytes [220]. Mechanistically, A2AR blockade with CPI-444 remarkably reduces the expression of PD-1 and LAG-3 on activated CD8+ effector T cells. Moreover, co-blockade of A2AR and A2BR has been shown to enhance CAR-T cell cytokine secretion, proliferation, cytotoxicity, and activation in vivo [221]. Together, these results highlight the translational potential of combining adenosine pathway inhibition with ACT, a strategy that could both improve ACT efficacy and expand its applicability to a wider range of malignancies.

## Future trends in the adenosinergic pathway

### Informative patient-selective strategies

A major challenge in advancing adenosine-targeting therapies is identifying cancers with significant adenosine-driven signaling and selecting patients most potentially to benefit from these treatments. Ideally, direct measurement of extracellular adenosine concentrations in the TME would enable the identification of cancers with substantial adenosine signaling. Due to the extremely short half-life of adenosine (t_1/2_ approximately 10 s) [222], quantifying tumor adenosine levels at scale is difficult, necessitating the use of molecular surrogates.

Generally, pharmacological inhibition of the eADO pathway often triggers responses in cancers enriched with adenosinergic components, such as renal cell carcinoma, colorectal cancer, pancreatic cancer, and lung cancers​. However, direct correlations between adenosinergic component expression and therapeutic efficacy have yet to be validated. For example, responses to these agents have been observed in cancers with low baseline adenosinergic signaling, such as advanced prostate cancer. In a clinical trial of ciforadenant in RCC, pretreatment tumor CD73 expression levels did not correlate with clinical response [223].

Nevertheless, certain tumor-intrinsic features drive adenosine pathway activity and may help identify tumors responsive to treatment. Oncogenic mutations, such as *TP53, EGFR*, and *RAS*, can upregulate CD73 expression, promoting adenosine-mediated immunosuppression and enhancing tumor sensitivity to CD73 inhibition [86]. Likewise, hypoxic gene signatures and tissue-repair processes, including EMT and TGF-β signaling, also increase CD73 expression [86]. Emerging evidence identifies the A2AR/PKA/mTORC1 axis as a primary adenosine-mediated pathway suppressing both peripheral T cells and tumor-infiltrating lymphocytes. This suggests that p-CREB (a PKA activation marker) and p-S6 (an mTORC1 activity indicator) could serve as dual pharmacodynamic and efficacy biomarkers for adenosine-targeted therapies [76]. Additionally, soluble CD73, shed from cell surfaces into the bloodstream, is being explored as a systemic biomarker. Elevated soluble CD73 levels in metastatic melanoma patients undergoing immunotherapy correlate with worse outcomes [224]. If validated, high soluble CD73 could identify patients with active adenosine-mediated immunosuppression, marking them as potential candidates for adenosine-targeted therapies.

Transcriptional profiles provide an alternative means of identifying patients with adenosine-rich tumors. Fong et al. [171] developed the "AdenoSig" gene signature by stimulating normal human peripheral blood mononuclear cells (PBMCs) with A2AR agonists (*CXCL1, 2, 3, 4, 5, ILB, IL1B,* and *PTGS2*) in vitro*.* This gene set was subsequently evaluated in pretreatment tumor biopsies, revealing a correlation between AdenoSig expression and response to A2AR inhibitors. Patients with high AdenoSig expression (AdenoSig^hi^) in pretreatment biopsies demonstrated more pronounced tumor regression and longer progression-free survival (PFS). This suggests that AdenoSig^hi^ patients may respond better to A2AR antagonists in combination with anti-PD-1/PD-L1 therapies, compared to those receiving anti-angiogenesis treatments. Overall, this research demonstrates the potential of the AdenoSig signature to predict responses to the A2AR antagonist ciforadenant in RCC, positioning it as a valuable tool for selecting patients likely to benefit from adenosine-targeting therapies.

In contrast, the adenosine signaling score, developed by Sidders et al*.* [172], consists of a gene cluster (*PPARG, CYBB, COL3 A1, FOXP3, LAG3, APP, CD81, GPI, PTGS2, CASP1, FOS, MAPK1, MAPK3,* and *CREB1*) reflecting adenosine activity in human cancers. This signature directly correlates with baseline adenosine levels in vivo, which are reduced following A2AR blockade in a murine syngeneic model. Notably, the adenosine signaling score was identified as a negative predictor of OS and PFS in data from The Cancer Genome Atlas. Moreover, baseline adenosine signaling scores were inversely correlated with response to anti-PD-1 therapy in published cohorts. These findings suggest that the adenosine signaling score could inform patient selection for immunotherapy and adenosine pathway modulation. Despite sharing only a single common gene, AdenoSig and the adenosine signaling score exhibit a strong correlation across four cancer types (RCC, NSCLC, prostate cancer, and melanoma) [225]. The biological relationship between these two signatures indicates that both signatures could be useful for clinical trial screening.​ Further studies are needed to explore additional mechanisms influencing immunotherapy sensitivity and to refine this signature by incorporating a broader transcript panel.

The complexity of the TME and the context-specific function of adenosine preclude the establishment of a single, definitive biomarker. Efforts are underway to pinpoint molecular surrogates for adenosine-rich tumors. Emerging data indicate that gene expression signatures, such as AdenoSig and the adenosine signaling score, are promising due to their association with clinical outcomes. Integrating these genetic signatures with other identified biomarkers may enhance the ability to identify patients most likely to benefit from targeting the adenosine pathway.

### Considerations for eADO-targeting agents

In recent years, several pharmacological inhibitors targeting the adenosinergic pathway have been developed, showing promising clinical activity both as single agents and in combination therapies. Many A2AR antagonists were originally designed for neurologic disorders, which indicates that these small-molecule drugs possess the ability to cross the blood-brain barrier (BBB) [226–229]. However, the high expression of CD73 in non-malignant tissues may lead to on-target toxicities when anti-CD73 mAbs inhibit CD73 function in these tissues. Therefore, further research is needed to optimize the development of these agents to achieve better penetration and distribution within the TME while minimizing effects on peripheral tissues. For instance, the A2AR antagonist EOS100850 has been specifically designed to have minimal BBB penetration while maintaining potent activity within the TME [230]. In an effort to enhance therapeutic efficacy, Ploeg et al. [231] developed a novel tetravalent bispecific antibody (bsAb), named bsAb CD73xEGFR. This bsAb not only blocks the CD73/adenosine immune checkpoint but also targets EGFR, counteracting multiple oncogenic pathways associated with both EGFR and CD73.

Improved drug delivery systems may also be crucial for enhancing the bioavailability and effectiveness of adenosine-targeting therapies. Nanotechnology-based systems have demonstrated significant tumor penetration and extended blood circulation, indicating that immunomodulatory nanomedicines could overcome existing drug delivery limitations [232–238]. Our previous work successfully fabricated small silver nanoparticles (S-AgNPs) [239] and immunostimulant nanobombs (Apt@SCH@BPs) [240] that showed superior antitumor effects and better tumor targeting. Additionally, Chen et al. [241] developed nanoscale coordination particles (AmGd-NPs) composed of gadolinium (Gd) and a small molecular CD73 inhibitor (AmPCP). These nanoparticles effectively inhibit the conversion of extracellular ATP to adenosine, driving a pro-inflammatory TME that enhances DC maturation. Furthermore, Mao et al. [158] created ROS-producing nanoparticles loaded with CD39/CD73 inhibitors (ARL) to prevent ATP degradation and reprogram the immunogenic landscape within tumors. Nanoparticles have also proven to be a versatile platform for silencing RNA (siRNA) and microRNA (miRNA) -based therapies targeting purinergic signaling [242]. For instance, the delivery of CD73 siRNA via nanoparticles to melanoma cells successfully downregulated CD73 expression, enhancing T-cell-specific immunity and improving the efficacy of ICB therapies [243]. Similarly, SPION-CL-TAT nanoparticles loaded with anti-PD-1 and A2AR siRNAs efficiently delivered siRNA to tumor-derived T cells and suppressed the expression of both A2AR and PD-1 ex vivo [203]. These findings underscore the potential of immune-nanoactivators in modulating the adenosine pathway and offer a novel therapeutic paradigm for cancer treatment.

### ADA: a novel immunotherapy target?

In addition to CD73 and CD39, various enzymatic pathways contribute to adenosine production, rendering the complete inhibition of adenosine synthesis within the TME an unrealistic goal. Extracellular adenosine interacts with four distinct G-protein coupled receptors (A1, A2A, A2B, and A3), exerting both anti-tumor [244–246] and pro-tumor [176, 247, 248] effects. The comprehensive impact of adenosine accumulation on tumor progression remains poorly understood. Notably, A2AR deletion has been shown to significantly upregulate CD73 expression, suggesting a potential auto-regulatory loop that tumors may exploit to sustain adenosine production [188]. Rather than conventional adenosine-targeted therapies, inhibiting adenosine-lowering enzymes may offer a more promising strategy.

Adenosine deaminase (ADA) catalyzes the conversion of adenosine to inosine through a deamination reaction. In humans, ADA exists in two isoforms, ADA1 and ADA2 [249]. The monomeric ADA1 is primarily involved in the degradation of intracellular adenosine and deoxyadenosine, while ADA2, a dimeric enzyme found in serum, mainly catalyzes the deamination of adenosine. ADA2 exhibits optimal activity in weakly acidic environments, such as those found in hypoxic conditions [250]. The K_m_ value of ADA2 is 100 times higher than that of ADA1 [250], indicating that ADA2 is more relevant in the metabolism of pathologically elevated adenosine levels rather than under steady-state conditions.

Preclinical studies by Wang et al. [251] demonstrated that ADA2 expression is associated with improved survival in patients with cancers. To assess the potential of ADA2 as an anticancer immunotherapy, the group engineered a PEGylated form of ADA2 to extend its systemic exposure. PEGylated ADA2 (PEGADA2) treatment suppressed tumor progression in an enzyme activity-dependent manner, modulating immune responses. Similarly, PEG-ADA, an FDA-approved enzyme replacement therapy for children with severe combined immunodeficiency (SCID), has been shown to alleviate adenosine-mediated suppression of CD8+ T cells and enhance responses to anti-PD-1 therapy [252]. Other studies have highlighted inosine's role in overcoming tumor-imposed metabolic constraints on T cells. Inosine serves as an alternative carbon source for effector T cells in glucose-deprived environments, supporting their growth and function within the nutrient-limited TME [253]. Additionally, inosine influences CAR-T cell metabolism and epigenetic stemness programming [254], and has been found to regulate tumor-intrinsic immunogenicity and modulate immunotherapy sensitivity [255]. These findings underscore the potential of targeting ADA as a novel cancer immunotherapy, offering a viable strategy to overcome resistance to conventional treatments.

## Conclusions

Adenosine has emerged as a key player in cancer biology and oncology due to its potent immunosuppressive effects and critical role in promoting tumorigenesis. Targeting the adenosine-mediated signaling pathway offers a promising approach to enhance immunotherapy efficacy and overcome resistance to established cancer therapeutics. Selective inhibitors that block adenosine generation or its receptor binding have been developed, demonstrating significant tumor growth inhibition in murine models. Over 30 clinical trials are currently in Phase 1, with numerous preclinical agents under investigation, reflecting the growing interest in targeting adenosinergic pathways for cancer therapy and the transition toward clinical validation. Moreover, combining adenosine pathway blockade with immune checkpoint inhibition and adoptive cellular therapy has shown synergistic effects and favorable tolerability in preclinical paradigms. To fully realize the therapeutic potential of adenosine-targeting strategies, future research must address several key challenges.

Notably, given the compensatory mechanisms within the adenosine pathway in response to single-node blockade, investigating the feasibility of concurrently targeting multiple loci within the pathway is crucial. Furthermore, significant questions persist regarding the efficacy of existing agents and the identification of biomarkers capable of predicting patient responses to adenosine-targeted therapies. Additionally, evaluating whether modulation of adenosine-lowering enzymes can improve therapeutic outcomes remains a critical consideration. Addressing these issues should be a priority to advance adenosine-targeting agents for broader clinical application.

## Data Availability

No datasets were generated or analysed during the current study.

## Abbreviations

A1R: Adenosine 1 receptor
A2AR: Adenosine 2A receptor
A2BR: Adenosine 2B receptor
A3R: Adenosine 3 receptor
ABC: ATP-binding cassette
AC: Adenylate cyclase
ACT: Adoptive cellular therapy
ADA: Adenosine deaminase
ADCP: Antibody-mediated cellular phagocytosis
ADP: Adenosine diphosphate
ADPR: Adenosine diphosphate ribose
AEs: Adverse events
AK: Adenylate kinase
AMP: Adenosine monophosphate
APs: Alkaline phosphatases
ATP: Adenosine triphosphate
BBB: Blood-brain barrier
BLCA: Bladder urothelial carcinoma
BRCA: Breast invasive carcinoma
bsAb: Bispecific antibody
CAFs: Cancer-associated fibroblasts
cAMP: Cyclic adenosine monophosphate
CAR: Chimeric antigen receptors
CD73: Ecto-5′-nucleotidase
CESC: Cervical squamous cell carcinoma
CHOL: Cholangiocarcinoma
CNTs: Concentrative nucleoside transporters
COAD: Colon adenocarcinoma
COX2: Cyclooxygenase 2
CRC: Colorectal cancer
CREB: CAMP-response element binding protein
CR: Complete response
CSF2: Colony stimulating factor 2
CTLA4: Cytotoxic T-lymphocyte antigen 4
CXCL12: C-X-C motif chemokine ligand 12
DCs: Dendritic cells
DCR: Disease control rate
eADO: Extracellular adenosine
EMT: Epithelial-mesenchymal transition
ENPPs: Ectonucleotide pyrophosphatase/phosphodiesterases
E-NTPDases: Ectonucleoside triphosphate diphosphohydrolases
ENTs: Equilibrative nucleoside transporters
Epac: Exchange proteins directly activated by cAMP
ESCA: Esophageal carcinoma
FASL: Fas ligand
FDA: The US Food and Drug Administration
GBM: Glioblastoma multiforme
GPCRs: G-protein-coupled receptors
HCC: Hepatocellular carcinoma
HNSC: Head and neck squamous cell carcinoma
HIF1α: Hypoxia-inducible factor 1α
ICI: Immune checkpoint inhibitor
ICB: Immune checkpoint blockade
IDO: Indoleamine 2,3-dioxygenase
IFN-γ: Interferon-γ
IL-1: Interleukin 1
IL-10: Interleukin 10
IL-12: Interleukin 12
IL-13: Interleukin 13
IL-18: Interleukin 18
IL-2: Interleukin 2
IL-4: Interleukin 4
KICH: Kidney chromophobe carcinoma
KIRC: Kidney renal clear cell carcinoma
KIRP: Kidney renal papillary cell carcinoma
LIHC: Liver hepatocellular carcinoma
LUAD: Lung adenocarcinoma
LUSC: Lung squamous cell carcinoma
mCRPC: Metastatic castrate-resistant prostate cancer
MDSCs: Myeloid-derived suppressor cells
miRNA: MicroRNA
mTORC1: Mechanistic target of rapamycin complex 1
NDPK: Nucleoside diphosphate kinase
NET: Neutrophil extracellular trap
NK: Natural killer cell
NLRP3: NLR family pyrin domain-containing protein 3
NSCLC: Non-small cell lung cancer
NF-κB: Nuclear factor‑κB
OS: Overall survival
ORR: Objective response rate
P2XR: P2X receptor
P2YR: P2Y receptor
PAAD: Pancreatic adenocarcinoma
PAPs: Prostatic acid phosphatases
PBMCs: Peripheral blood mononuclear cells
PCPG: Pheochromocytoma and Paraganglioma
PD1: Programmed cell death protein-1
PDAC: Pancreatic ductal adenocarcinoma
PFS: Progression-free survival
PI3K: Phosphatidylinositol-3-kinase
PKA: Protein kinase A
PNP: Purine nucleoside phosphorylase
PRAD: Prostate adenocarcinoma
PRF1: Perforin 1
PR: Partial response
READ: Rectum adenocarcinoma
ROS: Reactive oxygen species
SCID: Severe combined immunodeficiency
siRNA: Silencing RNA
STAD: Stomach adenocarcinoma
STAT3: Signal transducer and activator of transcription 3
SD: Stable disease
TCR: T cell receptor
Teff: Effector T cell
Tex: T cell exhaustion
TGF-β: Transforming growth factor β
THCA: Thyroid carcinoma
THYM: Thymoma
TLR: Toll-like receptors
TME: The tumor microenvironment
TNAPs: Tissue-non-specific alkaline phosphatases
TNBC: Triple-negative breast cancer
TNF: Tumor necrosis factor
TNF-α: Tumor necrosis factor α
Treg: Regulatory T cell
UCEC: Uterine corpus endometrial carcinoma
VEGF: Vascular endothelial growth factor
